# Overcoming resistance to PD-1 and CTLA-4 blockade mechanisms and therapeutic strategies

**DOI:** 10.3389/fimmu.2025.1688699

**Published:** 2025-10-03

**Authors:** Xiaodong Wang, Jing He, Gouping Ding, Yixuan Tang, Qianqian Wang

**Affiliations:** Department of Oncology, Zhuzhou Hospital Affiliated to Xiangya School of Medicine, Central South University, Zhuzhou, China

**Keywords:** PD-1/CTLA-4 blockade resistance, tumor microenvironment, T cell exhaustion, immunotherapy combinations, alternative checkpoints, predictive biomarkers

## Abstract

Immune checkpoint inhibitors (ICIs) targeting PD-1 and CTLA-4 have achieved groundbreaking clinical success in multiple cancers; however, a large proportion of patients experience primary or acquired resistance. This review synthesizes the complex mechanisms underlying resistance to PD-1/CTLA-4 blockade and surveys emerging strategies to overcome them. Resistance arises from multifaceted interactions among tumor-intrinsic alterations (e.g., epigenetic silencing of antigen presentation machinery via EZH2/PRC2, oncogenic pathway–driven upregulation of PD-L1, genetic loss of IFNγ pathway components such as JAK1/2 or B2M), immune cell dysfunction (e.g., T cell exhaustion with co-expression of inhibitory receptors including PD-1, TIM-3, and LAG-3, metabolic and epigenetic T cell reprogramming, suppressive regulatory T cells), and stromal microenvironmental factors (e.g., hypoxia-inducible factors, immunosuppressive metabolites like IDO-mediated kynurenine, tumor-associated macrophages and MDSCs, aberrant angiogenesis). To counteract these diverse resistance mechanisms, a spectrum of novel therapeutic approaches is under development. Mechanism-targeted monotherapies include agents that restore tumor immunogenicity (e.g., epigenetic modulators to upregulate MHC expression), reinvigorate exhausted T cells (e.g., blockade of alternative checkpoints such as LAG-3), and reprogram the suppressive tumor microenvironment (e.g., inhibitors of immunosuppressive myeloid pathways). In parallel, rational combination therapies are being explored, pairing ICIs with chemotherapy (to induce immunogenic cell death and enhance T cell infiltration), molecularly targeted drugs (to disrupt oncogenic immune-evasion signals), or immune modulators (e.g., IL-2 or IL-18 variants to boost effector T cell function). Furthermore, emerging predictive biomarkers and machine learning-based signatures (e.g., soluble checkpoint levels, inflammatory indices, tumor transcriptomic scores) are improving the ability to anticipate ICI resistance and guide personalized escalation of therapy. Overall, this synthesis highlights the recent insights into resistance biology and promising avenues to extend the durable benefits of PD-1/CTLA-4 blockade to a larger proportion of patients.

## Graphical Abstract

Mechanisms and therapeutic strategies for overcoming PD-1/CTLA-4 blockade.
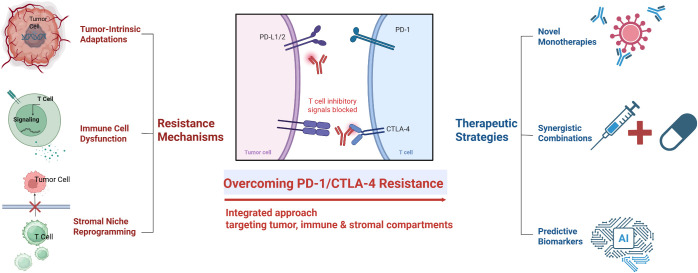

## 1 Introduction

Cancer immunotherapy, particularly blockade of the immune checkpoints PD-1 and CTLA-4, represents a paradigm shift in oncology, yielding durable remissions in previously untreatable advanced malignancies (1–3). CTLA-4, expressed primarily on T cells (including activated T cells and regulatory T cells), functions early in the immune response within lymphoid organs by outcompeting the costimulatory receptor CD28 for B7 ligands (CD80/CD86) on antigen-presenting cells. This interaction attenuates T cell priming (4–6). PD-1, expressed on activated T cells (as well as on B cells and myeloid cells), delivers inhibitory signals upon engaging its ligands PD-L1 or PD-L2, predominantly in peripheral tissues and the tumor microenvironment (TME), leading to T cell functional exhaustion (7, 8). Monoclonal antibodies targeting CTLA-4 (e.g., ipilimumab) or PD-1/PD-L1 (e.g., nivolumab, pembrolizumab, atezolizumab) release these brakes, thereby reinvigorating anti-tumor immunity and demonstrating remarkable clinical efficacy across diverse cancer types, including melanoma, non-small cell lung cancer (NSCLC), renal cell carcinoma, and mismatch repair–deficient cancers (1, 9–12). Notably, combined blockade of CTLA-4 and PD-1 yields superior response rates compared to monotherapy, albeit with increased toxicity (13).

Despite these successes, a substantial proportion of patients fail to respond initially (primary resistance) or relapse after an initial response (acquired resistance) (14). Even in immunogenic tumors such as melanoma or NSCLC, objective response rates to PD-1/PD-L1 monotherapy remain only approximately 20%–45%, and combination anti-CTLA-4/PD-1 therapy, while improving responses, still leaves more than half of patients without durable benefit (15–17). This resistance remains a major barrier to improving long-term survival. The underlying mechanisms are extraordinarily complex and heterogeneous, involving dynamic interactions among tumor cells, immune cells, and other stromal components. Tumor cells can evade immune elimination through loss or downregulation of antigen-presenting machinery (e.g., β2-microglobulin or MHC class I), upregulation of alternative immune inhibitory ligands, and activation of oncogenic pathways that foster an immune-hostile milieu (18–20). Simultaneously, tumor-infiltrating lymphocytes (TILs) may become functionally impaired or “exhausted,” characterized by co-expression of multiple inhibitory receptors, metabolic insufficiency, and epigenetic fixation in a hypofunctional state (21).

Furthermore, the surrounding TME actively suppresses immunity through tumor-induced conditions such as hypoxia (stabilizing HIF-1α); accumulation of immunosuppressive metabolites such as kynurenine (via indoleamine 2,3-dioxygenase, IDO), which promote regulatory T cells and exhaust effector T cells; recruitment of suppressive myeloid cells (tumor-associated macrophages, myeloid-derived suppressor cells) and fibroblasts; and aberrant vasculature that limits effector T cell infiltration (22–24). While significant progress has been made in identifying individual resistance pathways, a comprehensive understanding of how these diverse mechanisms intersect is critical for devising effective strategies to overcome resistance.

## 2 PD-1 and CTLA-4: physiological roles in immune regulation and cancer

To set the stage for this discussion, we first review the normal physiological roles of PD-1 and CTLA-4 in immune regulation, as well as how tumors exploit these pathways.

PD-1 and CTLA-4 are negative regulators, each serving distinct roles in the immune response. Cytotoxic T-Lymphocyte Antigen 4 (CTLA-4) is upregulated on T cells shortly after initial activation in lymphoid organs and is expressed constitutively on regulatory T cells (Tregs) (25). By competing with the co-stimulatory receptor CD28 for binding to B7-1/B7-2 (CD80/CD86) on antigen-presenting cells, CTLA-4 reduces the amplitude of early T cell activation (5, 26). This mechanism helps maintain self-tolerance and prevents autoimmunity during T cell priming. In the tumor context, however, this checkpoint can be subverted, as tumors may induce strong CTLA-4 signaling to limit the priming of tumor-specific T cells. Tregs within the tumor can also use CTLA-4 to sequester CD80/86 and deliver inhibitory signals, thereby dampening anti-tumor T cell responses (27, 28). Notably, germline disruption of CTLA-4 in mice results in fatal lymphoproliferation, underscoring its critical role in immune homeostasis (29). Blocking CTLA-4 with antibodies (e.g., ipilimumab) enhances the priming and expansion of effector T cells but also carries the risk of broad immune activation, accounting for the immune-related toxicities observed clinically (30, 31).

**Figure 1:**
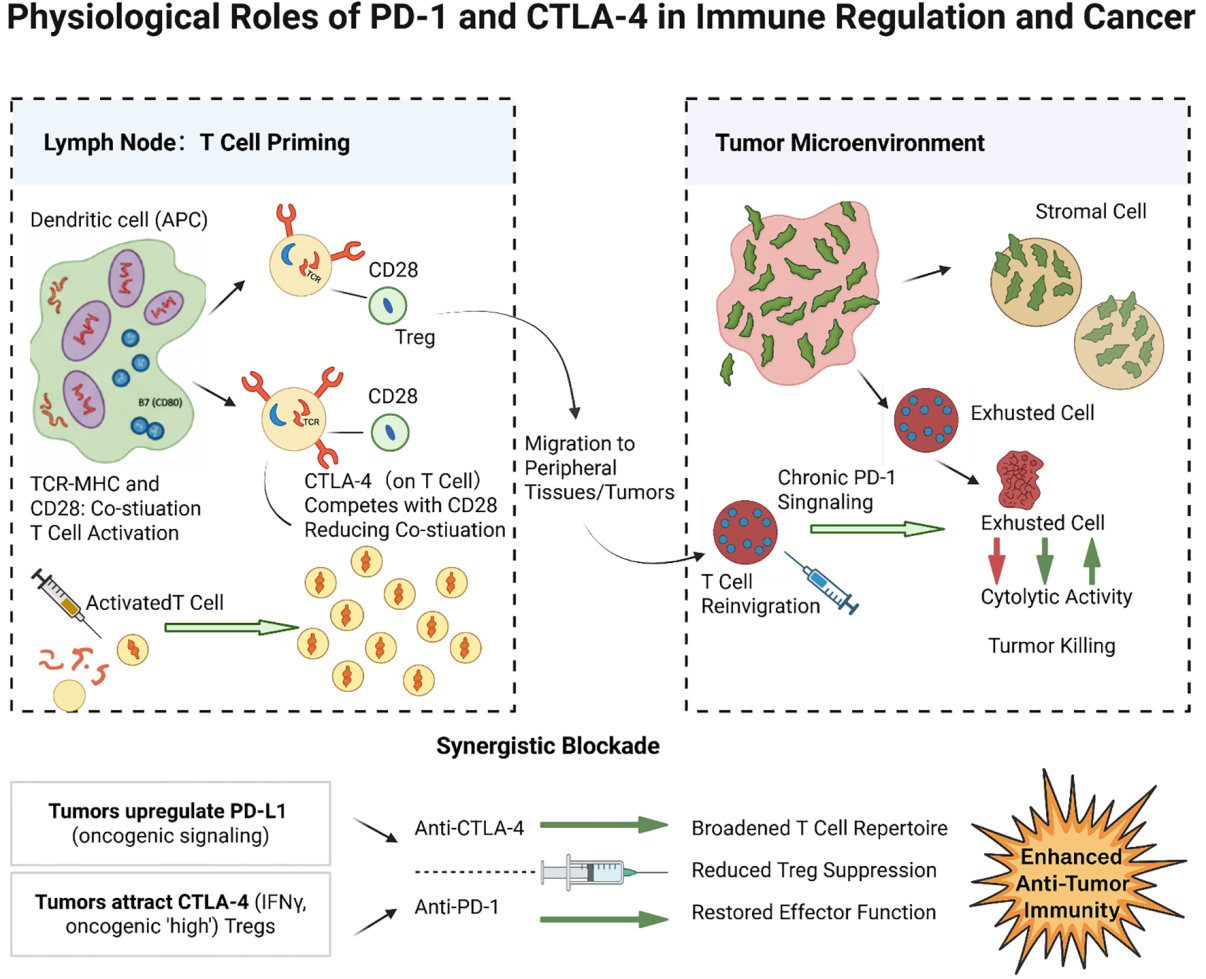
Physiological roles of PD-1 and CTLA-4 in immune regulation and cancer. CTLA-4 on activated T cells (and Tregs) in lymphoid organs competes with CD28 for B7 ligands on dendritic cells, thereby reducing co-stimulation and limiting T cell activation. In tumors, abundant CTLA-4 activity (for example, from infiltrating Tregs) curtails the priming of anti-tumor T cells. PD-1 on tumor-infiltrating T cells binds PD-L1/PD-L2 on tumor or stromal cells in the TME, delivering inhibitory signals (via SHP2-mediated pathways) that exhaust T cell effector functions. This chronic PD-1 signaling leads to reduced cytokine production and killing capacity in T cells, contributing to tumor immune escape. Blockade of CTLA-4 predominantly acts at the priming phase (expanding the pool of tumor-reactive T cells), whereas blockade of PD-1 acts at the effector phase (releasing brakes on T cells to attack tumor cells in the TME). Tumors often upregulate PD-L1 (e.g., via IFNγ or oncogenic signaling) and attract CTLA-4^high^ Tregs to exploit these checkpoints. Blocking PD-1 and CTLA-4 can thus synergistically activate immune responses: anti-CTLA-4 broadens the T cell repertoire and reduces Treg-mediated suppression, while anti-PD-1 restores the function of exhausted T cells in the tumor.

Programmed Cell Death 1 (PD-1) is predominantly expressed on T cells following chronic antigen exposure, and, to a lesser extent, on B cells as well as NK and myeloid cells. PD-1 is a key mediator of peripheral tolerance: engagement by its ligands PD-L1 or PD-L2—often expressed on tumor cells, stromal cells, or APCs in peripheral tissues—delivers an inhibitory signal that reduces T cell proliferation, cytokine production, and cytolytic activity (32). This mechanism normally helps resolve immune responses while preventing autoimmunity in peripheral tissues. In cancer, PD-1 is often highly expressed on TILs due to persistent tumor antigen exposure, while its ligands PD-L1 and PD-L2 can be upregulated on tumor or immune cells in the TME—for instance, PD-L1 is frequently induced by tumor cell oncogenic signaling or IFNγ exposure (33, 34). The PD-1/PD-L1 interaction in the TME induces a state of T cell “exhaustion,” characterized by impaired effector function and sustained expression of multiple inhibitory receptors (35, 36). This exhaustion is partially reversible: antibodies blocking PD-1 can reinvigorate these T cells, restoring their ability to proliferate and kill tumor cells (37, 38). However, fully exhausted T cells may not fully recover even with PD-1 blockade, particularly if additional inhibitory pathways (such as TIM-3, LAG-3) remain active or if the TME lacks supportive factors (39, 40). **Figure 1** illustrates the distinct roles of these checkpoints: CTLA-4 primarily regulates T cell activation in lymph nodes, whereas PD-1 primarily suppresses T cell activity in peripheral tissues and tumors. Tumors exploit both pathways—inducing CTLA-4 signaling to restrict T cell priming and upregulating PD-L1 to disable effector T cells—to evade immune destruction.

## 3 Mechanisms of resistance to PD-1/CTLA-4 blockade

**Figure 2:**
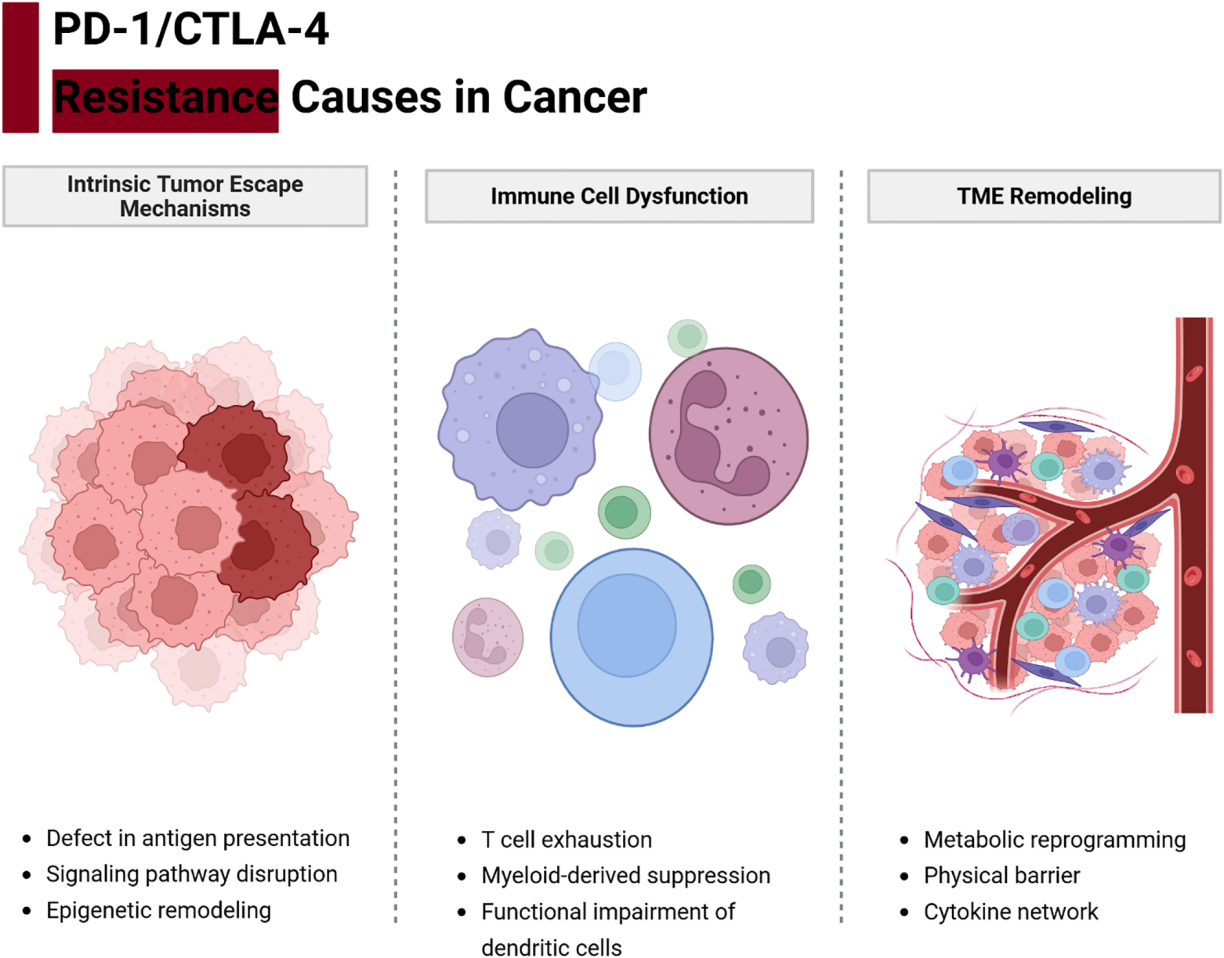
Integrated network of resistance mechanisms to PD-1/CTLA-4 blockade. Tumor cells, immune cells, and stromal elements form an interconnected network that can undermine immune checkpoint therapy. Tumor-intrinsic factors (red) such as impaired antigen presentation (e.g., MHC-I loss, B2M or JAK mutations) and oncogene-driven expression of immunosuppressive molecules (e.g., PD-L1 via EGFR/MAPK signaling) prevent effective T cell recognition. Immune cell dysfunction (blue) arises when tumor-reactive T cells become exhausted (marked by multiple inhibitory receptors and metabolic dysregulation) or when immunosuppressive cells like Tregs dominate. Stromal and microenvironmental factors (green)—including hypoxia, immunosuppressive metabolites (e.g., adenosine, kynurenine), suppressive tumor-associated macrophages and MDSCs, cancer-associated fibroblasts, and abnormal vasculature—create physical and chemical barriers to immune attack. These components reinforce one another: for example, tumors with loss of antigen presentation recruit more Tregs and MDSCs, and an immunosuppressive stroma induces more T cell exhaustion. Effective strategies to overcome resistance must therefore address multiple nodes of this network.

As shown in **Figure 2**, resistance to PD-1/CTLA-4 blockade arises from a multifaceted interplay among tumor-intrinsic adaptations, immune cell dysfunction, and stromal microenvironment reprogramming (14, 41, 42). These mechanisms often coexist and reinforce one another, creating an immunosuppressive network that diminishes the efficacy of ICIs (43).

### 3.1 Tumor-intrinsic oncogenic adaptations and immune evasion

**Figure 3:**
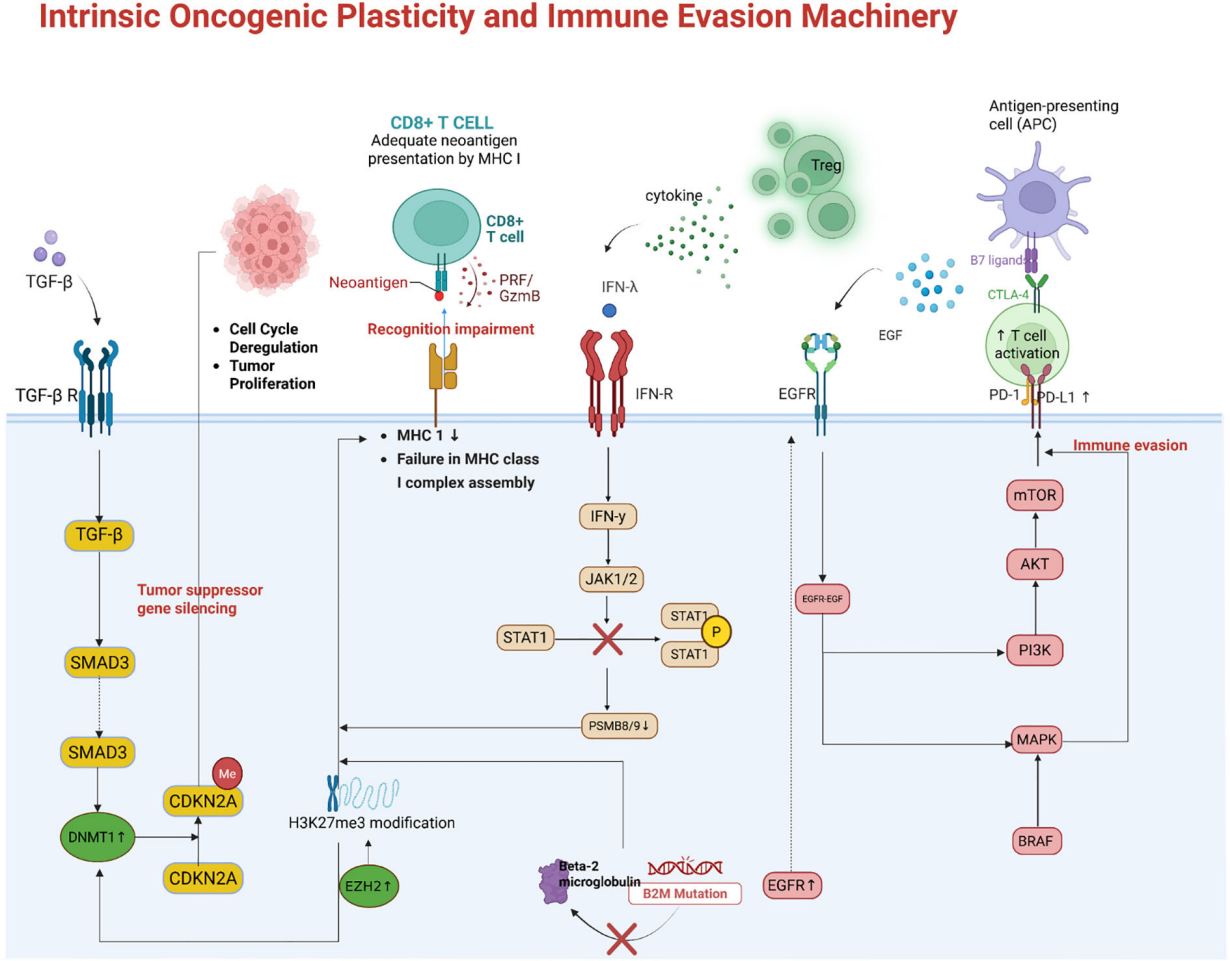
Tumor-intrinsic mechanisms of immune evasion. Epigenetic silencing of antigen presentation by EZH2/PRC2. Through trimethylation of H3K27, PRC2 coordinately represses genes encoding MHC-I and antigen-processing machinery (APM). This results in low surface MHC class I on tumor cells, preventing cytotoxic T lymphocyte recognition. Oncogenic signaling-driven upregulation of immunosuppressive ligands. Tumors with activated EGFR/MET or other pathways can overexpress PD-L1 and other inhibitory molecules, directly dampening T cell activity. For example, MET amplification in NSCLC induces PD-L1 expression, contributing to resistance to TKI and PD-1 therapy. Genetic defects in interferon and antigen presentation pathways. Loss-of-function mutations in JAK1/2 render tumor cells unresponsive to IFNg, so they fail to upregulate MHC and immune effector genes upon T cell attack. Similarly, B2M mutations abrogate cell-surface MHC-I expression. These tumor cells cannot be recognized by CD8 T cells, conferring resistance to ICIs.

Tumor cells exploit intrinsic genetic and epigenetic mechanisms to evade immune recognition, representing a major cause of resistance. One key strategy is the loss or silencing of antigen presentation machinery, which renders tumor cells “invisible” to cytotoxic T lymphocytes. For instance, tumors may acquire mutations in β2-microglobulin (B2M) or in components of the interferon signaling pathway (JAK1/2), leading to loss of MHC class I expression or insensitivity to IFNγ signals (44–48). Such defects disrupt the presentation of tumor antigens to T cells and are associated with primary resistance to PD-1/PD-L1 therapies in melanoma and other cancers. Similarly, copy number amplification of genes such as MDM2 and loss of PTEN have been linked to immune escape through both reduced antigen presentation and promotion of an inhibitory TME. Epigenetic modifications also play a pivotal role: the Polycomb Repressive Complex 2 (PRC2), via EZH2-mediated H3K27me3, can coordinately silence multiple genes in the antigen processing and presentation pathway (49, 50). As shown in **Figure 3**, EZH2-driven epigenetic silencing reduces MHC-I, TAP, and immunoproteasome subunit expression, thereby impairing tumor antigen display (51–53). This mechanism has been observed in tumors such as lymphomas and melanomas; accordingly, high EZH2 activity or low MHC expression correlates with poor responses to ICI (54). Targeting epigenetic regulators (e.g., with EZH2 or histone deacetylase inhibitors) can reverse this silencing and enhance tumor immunogenicity (55).

Another intrinsic resistance mechanism is the upregulation of alternative immune checkpoints and immunosuppressive molecules by tumor cells (56). Oncogenic signaling pathways often drive these changes. For example, activation of the EGFR, MAPK, or PI3K–AKT pathways in tumor cells can induce PD-L1 expression along with other immunoinhibitory factors (56–58). In NSCLC, acquired resistance to EGFR inhibitors (e.g., via MET amplification or secondary EGFR mutations) is accompanied by PD-L1 upregulation, creating a more suppressive TME (59, 60). **Figure 3** illustrates this process: oncogene-driven PD-L1 overexpression (for example, through the MET/HGF axis) directly blunts T cell activity and can cause resistance to subsequent PD-1 therapy (61, 62). Activation of the Wnt/β-catenin pathway is another example: it drives exclusion of T cells from the tumor, upregulates suppressive ligands, and has been associated with non-responsiveness to ICIs (63). Tumors with PTEN loss or PI3K–AKT activation not only proliferate unchecked but also secrete factors (such as VEGF and chemokines) that inhibit immune infiltration, thereby linking oncogenic mutations to immune evasion. Many of these oncogenic alterations are potential therapeutic targets (e.g., MET or VEGF inhibitors) Tumor-intrinsic resistance also results from genetic loss of interferon responsiveness (55). IFNγ released by T cells ordinarily upregulates antigen presentation and antiviral or cytotoxic genes in tumor cells (64). As shown in **Figure 3**, loss-of-function mutations in *JAK1* or *JAK2* (with concurrent deletion of the wild-type allele) abrogate IFNγ receptor signaling, preventing tumor cells from inducing MHC-I or PD-L1 in response to T cell attack. While loss of PD-L1 induction might seem beneficial, it actually reflects a state in which tumor cells cannot respond to immune cytokines at all, rendering them resistant to many immune effector mechanisms. *B2M* mutations have a similar effect by preventing surface expression of MHC-I. Clinically, such mutations have been identified in melanoma patients who progress despite PD-1 blockade, confirming their role in acquired resistance. Currently, there is no way to restore lost MHC or JAK function in tumors; therefore, alternative strategies—such as engaging NK cells (which can kill MHC-deficient cells) or using cell therapies that do not rely solely on tumor antigen presentation—are being explored to treat patients with these alterations.

In summary, tumor-intrinsic mechanisms of resistance include deficits in antigen presentation, upregulation of immune checkpoint ligands, and reduced sensitivity to immune cytokines, often driven by oncogenic events (65, 66). Together, these adaptations enable tumor cells to evade immune elimination despite PD-1/CTLA-4 blockade.

### 3.2 Immune cell functional imprint and checkpoint adaptation

**Figure 4:**
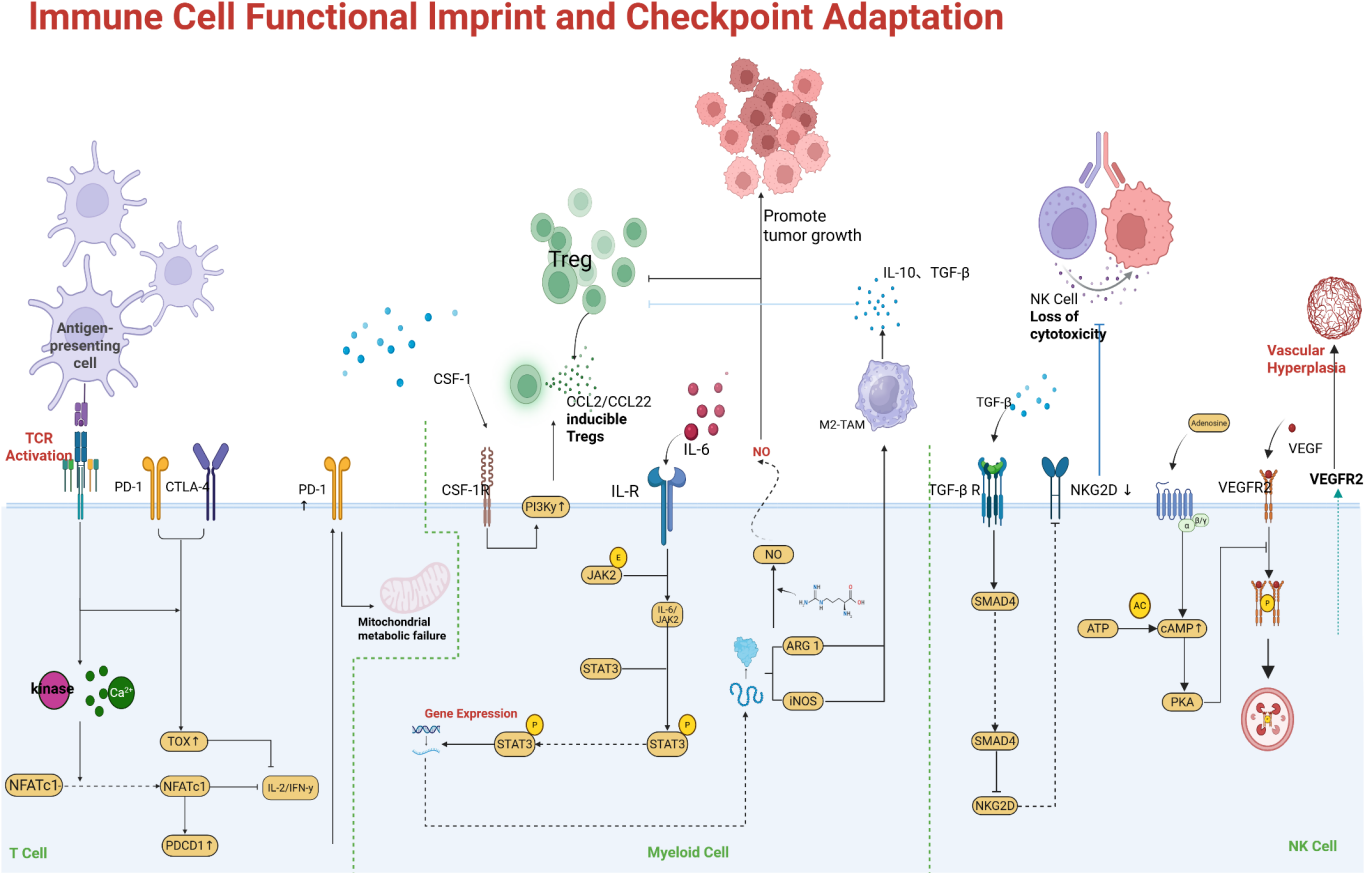
Immune cell dysfunction driving checkpoint resistance. Exhausted T cells co-express multiple inhibitory receptors (e.g., PD-1, TIM-3, LAG-3) and exhibit functional impairment. An example shown is the PD-1–Galectin-9–TIM-3 axis: Gal-9 on tumor or APCs can engage TIM-3 on T cells, typically inducing apoptosis. However, PD-1 on highly exhausted T cells can bind Gal-9 and form a PD-1/Gal-9/TIM-3 complex that prevents TIM-3-mediated cell death, allowing these dysfunctional T cells to persist. These persisting PD-1 cells have very low effector function, contributing to immune evasion. Synergy of multiple checkpoints: PD-1 and LAG-3 signals together drive deeper exhaustion. Concurrent blockade of LAG-3 (e.g., relatlimab) and PD-1 has been shown to synergistically restore T cell function, as evidenced by improved tumor control in preclinical models and higher response rates in melanoma patients versus PD-1 alone. Autocrine type I IFN signaling in T cells is also depicted as a factor reinforcing exhaustion (e.g., via lipid peroxidation and oxidative stress). Expansion of immunosuppressive cell populations. Tumors can induce proliferation and recruitment of Tregs and MDSCs that secrete inhibitory cytokines (IL-10, TGF-β) and express enzymes (IDO, arginase) that suppress effector T cells. An abundance of Tregs and MDSCs in the TME correlates with poor responses to ICIs. Therapies aiming to deplete Tregs (such as CTLA-4 or CCR4 antibodies) or reprogram macrophages (e.g., CSF-1R inhibitors) are being investigated to relieve this suppression.

Chronic exposure to tumor antigens and inhibitory signals can drive profound functional impairment in immune cells, particularly T cells, thereby blunting the efficacy of ICIs. T cell exhaustion is a hallmark of this dysfunction: tumor-infiltrating T cells initially activated against cancer can enter a progressive decline in effector function, characterized by reduced cytokine production, diminished cytotoxicity, and sustained high expression of inhibitory receptors such as PD-1, TIM-3, LAG-3, and TIGIT (67, 68). Exhausted T cells often co-express several of these checkpoints simultaneously, which helps explain why blocking a single checkpoint (e.g., PD-1 alone) may yield only partial reinvigoration. In addition, exhausted T cells undergo transcriptional and epigenetic reprogramming (e.g., upregulation of transcription factors TOX and NR4A, along with epigenetic changes that lock in an exhaustion phenotype) that may not be fully reversible (69). As the TME persists, exhaustion deepens: late-stage exhausted T cells lose proliferative capacity and may become resistant to IL-2 and other supportive signals (70). **Figure 4** highlights a notable interaction in exhausted T cells: the inhibitory receptor TIM-3 binds galectin-9 (Gal-9), and recent evidence indicates that Gal-9 can also form a complex with PD-1 on T cells. PD-1 binding to Gal-9 protects exhausted PD-1 TIM-3 T cells from Gal-9/TIM-3-induced apoptosis, allowing these highly dysfunctional cells to persist (71, 72). While this persistence might seem beneficial, it in fact maintains a pool of terminally exhausted T cells within the tumor that cannot effectively kill cancer cells but instead occupy the niche and secrete immunosuppressive cytokines. Thus, co-blockade of PD-1 and TIM-3, or disruption of PD-1–Gal-9 interactions, may be required to eliminate or reinvigorate this subset of T cells.

**Figure 5:**
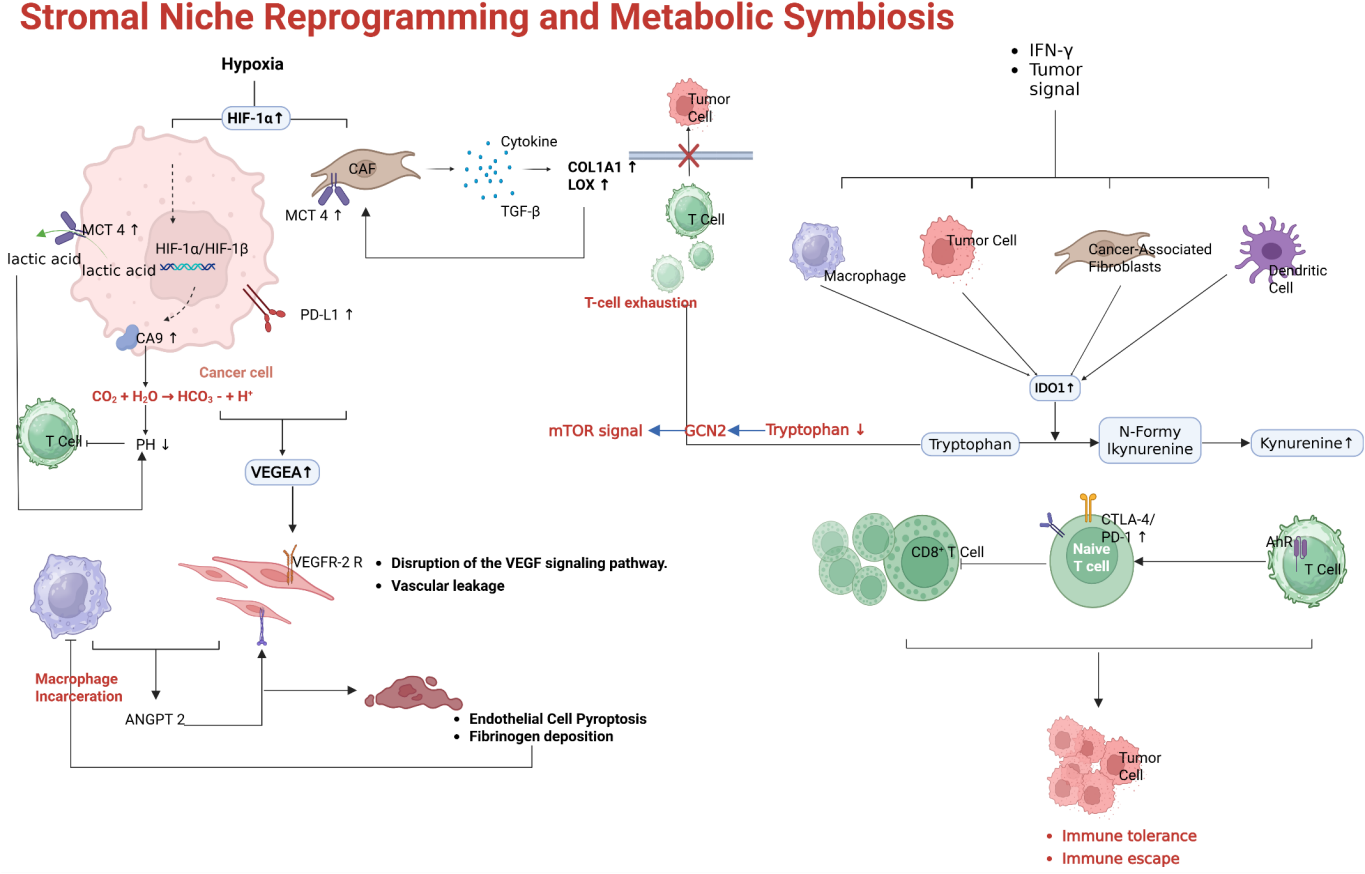
Stromal niche reprogramming fostering resistance. Tumor hypoxia and HIF-1α–driven immunosuppression. In low oxygen conditions, HIF-1α induces factors like VEGF (worsening vascular abnormality) and enzymes in TAMs such as legumain (LGMN) that promote M2 macrophage polarization. Hypoxia also elevates adenosine levels via CD39/CD73, which powerfully inhibits effector T and NK cells through A2A receptors. Myeloid cell-mediated suppression. Tumor-secreted chemokines (e.g., CCL2) recruit Ly6C monocytes that differentiate into MDSCs and immunosuppressive macrophages. These cells produce IDO, arginase, TGF-β, and IL-10, all of which curtail T cell function. IDO1-mediated tryptophan metabolism yields kynurenine, activating the aryl hydrocarbon receptor (AHR) in T cells and driving further immunosuppressive geneprograms. Therapies like CSF-1R inhibitors can deplete or re-polarize TAMs, and indeed CSF-1R blockade has reversed resistance in IDO-expressing tumors in mice. Abnormal vasculature and fibroblast barriers. Disorganized, leaky blood vessels in tumors lead to regions of hypoxia and limit Tcell infiltration. Cancer-associated fibroblasts form dense stroma and secrete extracellular matrix proteins, creating physical barriers; certain CAF subsets also express CD73 and produce adenosine or other immunosuppressive mediators. Agents like angiogenesis inhibitors or TGF-β pathway blockers can transiently normalize vessels or reduce stromal fibrosis, facilitating lymphocyte entry into tumors.

Another mechanism of immune adaptation is the concurrent upregulation of multiple inhibitory pathways. For example, LAG-3 is often co-expressed with PD-1 on exhausted CD8 T cells (73). **Figure 5** illustrates how LAG-3 and PD-1 signaling together impose a deeper state of exhaustion than either alone; preclinical models show that dual deficiency of LAG-3 and PD-1 unleashes markedly stronger T cell responses compared to single knockouts. In fact, LAG-3 can bind MHC-II on tumor cells or APCs, delivering a negative signal to T cells, and can also modulate the CD8 T cell compartment by affecting IL-2 production. Blocking LAG-3 (with relatlimab, for instance) alongside PD-1 has demonstrated improved outcomes in melanoma patients, validating this concept clinically (the combination of nivolumab and relatlimab achieved higher progression-free survival than nivolumab alone in advanced melanoma) (74). Other inhibitory receptors such as TIGIT (which binds CD155 on tumor cells or APCs) and VISTA contribute similarly; TIGIT on T cells dampens their activity and also suppresses dendritic cell function through engagement of CD155 (75). TIGIT blockade showed promise in early trials, but a large Phase 3 trial in NSCLC did not meet its endpoint, underscoring that optimal targeting (or patient selection, such as tumors with high CD155) is crucial. In exhausted T cells, chronic type I interferon (IFN-I) exposure in the TME has been implicated in reinforcing dysfunction: IFN-I can drive lipid peroxidation and metabolic stress in T cells, exacerbating their terminal exhaustion state (76). This suggests that modulating chronic inflammation in the TME (e.g., targeting IL-6 or IFN-I signaling) might help prevent T cells from becoming irreversibly exhausted.

Beyond CD8 T cell exhaustion, changes in other immune compartments also contribute to resistance. Regulatory T cells (Tregs), which strongly express CTLA-4 and often PD-1, accumulate in many resistant tumors and suppress effector T cells via cytokines (IL-10, TGF-β) and by depleting IL-2 (77). MDSCs and tolerogenic macrophages (often marked by ILT4, PD-L1, or SIRPα expression) can directly inhibit T cells through arginase, inducible nitric oxide synthase, and immune checkpoint ligand expression. Natural killer (NK) cells may also become dysfunctional or excluded from the tumor, and checkpoints such as NKG2A on NK cells (which binds HLA-E on tumor cells) can reduce NK-mediated killing. Notably, NKG2A has been identified as another inhibitory receptor whose expression in tumors may correlate with anti-PD-1 resistance. The interplay among these cells is complex. For instance, exhausted CD8 T cells may produce the chemokine CXCL13, which recruits additional Tregs and follicular helper T cells, reshaping the immune infiltrate.

Overall, immune cell–intrinsic mechanisms of resistance center on the failure of effector cells to maintain robust activity in the tumor. Checkpoint inhibitor therapy is less effective if T cells are too exhausted to respond or if other suppressive immune cells dominate. This understanding has prompted combination strategies targeting these pathways, such as combining PD-1 blockade with another checkpoint inhibitor (anti-LAG-3 or anti-TIGIT) to release multiple brakes or providing exogenous IL-2 to reactivated TILs to boost their proliferation and function. Additionally, therapies such as adoptive T cell transfer (engineered TILs or CAR T cells) can introduce fresh, non-exhausted immune effectors into the patient. Some of these approaches have shown early success; for example, transfusing autologous tumor-infiltrating lymphocytes after checkpoint blockade failure has achieved durable responses in melanoma patients (78).

### 3.3 Stromal niche reprogramming and metabolic symbiosis

The tumor microenvironment undergoes profound reprogramming to actively subvert immune attack. This involves not only cellular components (fibroblasts, endothelial cells, pericytes) but also acellular factors (oxygen tension, metabolites, extracellular matrix), all of which contribute to resistance against immunotherapy.

One major factor is hypoxia. Rapid tumor growth and abnormal vasculature lead to regions of low oxygen tension. Hypoxia stabilizes Hypoxia-Inducible Factor-1α (HIF-1α) in both tumor cells and infiltrating myeloid cells (79). As shown in **Figure 5**, HIF-1α drives the expression of multiple genes that suppress immune function. For example, in hypoxic TAMs, HIF-1α induces enzymes such as Legumain (LGMN), which polarize macrophages toward an M2-like, immunosuppressive phenotype (80). HIF-1α also upregulates VEGF and other factors that perpetuate abnormal angiogenesis, creating a vicious cycle of poor perfusion and ongoing hypoxia. Hypoxia in the TME directly affects T cells as well as low oxygen and nutrient deprivation impair T cell metabolism and effector function (81). Furthermore, hypoxic conditions promote the accumulation of adenosine in the extracellular space via CD39/CD73 ectonucleotidases on tumor and stromal cells (82). Adenosine is a potent immunosuppressive metabolite that signals through A2A receptors on T cells and NK cells to inhibit their activity. High intratumoral adenosine levels are associated with resistance to ICIs, and drugs targeting the adenosine pathway (CD73 inhibitors or A2A receptor antagonists) are being tested in combination with ICIs (83).

Another component of stromal reprogramming is the recruitment of immunosuppressive myeloid cells. Tumors often produce chemokines (CCL2, CXCL8, etc.) and growth factors (GM-CSF, M-CSF) that attract monocytes and neutrophils and skew their differentiation toward MDSCs and tumor-promoting macrophages (84). These myeloid cells inhibit T cell responses through multiple mechanisms: they secrete IL-10 and TGF-β, express checkpoint ligands (PD-L1, VISTA), and deplete nutrients essential for T cells (e.g., L-arginine via arginase). **Figure 5** highlights the role of myeloid-derived suppressor cells (MDSCs) and M2-polarized TAMs: tumor-derived factors such as IL-34, CSF-1, and midkine (MDK) drive the accumulation of MDSCs and M2 macrophages, which in turn release nitric oxide (NO) and reactive oxygen species that disable nearby T cells, and upregulate IDO1, which depleting tryptophan and produces kynurenine (85). Kynurenine (the product of IDO/TDO-mediated tryptophan metabolism) binds the aryl hydrocarbon receptor (AHR) in T cells and further induces immunosuppressive genes, reinforcing T cell dysfunction (86). In fact, tumors with high IDO1 expression were initially thought to be prime candidates for IDO inhibitor therapy combined with PD-1 blockade. While the first major trial of an IDO1 inhibitor (epacadostat) plus pembrolizumab in melanoma was surprisingly negative (showing no improvement over pembrolizumab alone) (87), the role of tryptophan metabolism in immunosuppression remains clear, and other approaches to target this pathway (e.g., alternative IDO/TDO inhibitors or AHR antagonists) are under investigation. Additionally, blocking recruitment pathways for MDSCs, such as the CCR2–CCL2 axis or CSF-1/CSF-1R signaling, has shown signs of restoring anti-tumor immunity in preclinical models.

Cancer-associated fibroblasts (CAFs) represent another stromal element that can mediate resistance (88). Certain subsets of CAFs express immune-inhibitory factors such as CXCL12 (which can form a barrier to T cells) and cell-surface proteins including FAP and PD-L2. Some CAFs express CD73 and generate adenosine, as mentioned above, while others produce ECM that increases interstitial pressure, physically impairing T cell infiltration. Recent studies have identified a specific fibroblast subpopulation linked to immunotherapy resistance via CD73-mediated adenosine production. Depleting or reprogramming CAFs (for example, with FAP-targeted therapies or TGF-β inhibitors that modulate fibroblast activation) can make the TME more permissive to immune cell penetration.

Leaky, disorganized blood vessels in tumors lead to poor oxygenation and hinder T cell trafficking (89). Certain therapies, such as low-dose or “metronomic” chemotherapy (e.g., low-dose gemcitabine) or anti-angiogenic drugs (VEGFR inhibitors) can transiently normalize vasculature, thereby increasing immune cell entry into tumors. Indeed, combining anti-angiogenics (such as bevacizumab or multi-kinase inhibitors such as lenvatinib targeting VEGFR) with ICIs has yielded improved response rates in certain settings (e.g., renal cell carcinoma, hepatocellular carcinoma), presumably by both relieving vascular constraints and modulating myeloid cell infiltration (90).

Finally, beyond these well-characterized factors, emerging research highlights that host’s systemic factors (the so-called *exposome*) and neuronal interactions can influence the tumor immune microenvironment (91–94). Chronic inflammation or microbiota dysbiosis due to diet, commensal microbes, or concomitant medications can set an immunological tone that impacts ICI response (95). For example, antibiotic use before or during ICI therapy has been associated with poorer outcomes in multiple cancers, likely by disrupting gut microbiota that support anti-tumor immunity. Conversely, fecal microbiota transplantation (FMT) from ICI-responding patients into refractory patients has converted non-responders to responders in early trials (95). The “exposome” also includes factors such as smoking, obesity, and infections or vaccinations, which can modulate immune responses to cancer (93). Meanwhile, a groundbreaking study in 2025 revealed that *cancer-induced nerve injury* within tumors promotes immunotherapy resistance: tumors invading nearby nerves cause neuronal damage, triggering an autonomous nerve response involving IL-6 and type I IFNs that establishes a chronic inflammatory, immunosuppressive milieu (94). This perineural niche inflammation skews the TME toward exhaustion and tolerance, and in patients with head and neck cancers and melanoma, the presence of nerve invasion correlated with anti-PD-1 failure (94). Intriguingly, blocking IL-6 signaling or even denervating tumors restored responsiveness in preclinical models. These findings broaden our understanding of resistance: not only tumor and immune cells but also systemic and neural factors contribute to an immunosuppressive network.

In summary, stromal and microenvironmental reprogramming—through hypoxia/HIF-1α pathways, metabolic suppression (IDO, adenosine), immunosuppressive myeloid and fibroblast activity, abnormal vasculature, and systemic metabolic and neural influences—collectively foster a tumor niche resistant to immune checkpoint blockade. Successful therapeutic strategies must therefore address these components.

## 4 Therapeutic strategies to overcome resistance

Resistance to PD-1/PD-L1 and CTLA-4 blockade can be countered by tailoring therapies to the underlying mechanism of immune evasion. Rather than classifying approaches as monotherapies or combinations, current strategies are best organized by the resistance mechanisms they target. Below, we discuss interventions aimed at (1) tumor-intrinsic resistance (e.g., impaired antigen presentation, oncogenic immune evasion) (2), immune cell dysfunction (e.g., T cell exhaustion, alternative checkpoints), and (3) the immunosuppressive tumor microenvironment (e.g., myeloid cells, metabolites, stroma). **Table 1** summarizes key mechanism-targeted monotherapies, and **Table 2** outlines rational combination regimens designed to overcome resistance.

**Table 1:** Summary of mechanism-targeted monotherapies to overcome PD-1/CTLA-4 resistance.

Category	Representative agent/approach	Mechanism of action	Key preclinical/clinical evidence	References
Tumor-intrinsic targets	Anti-CTLA-4 ADC	Targets CTLA-4+ tumor cells; delivers cytotoxin to eliminate immunosuppressive clones (e.g., those releasing sCTLA-4).	Preclinical: Selectively kills resistant CTLA-4–expressing GI cancer cells, countering sCTLA-4-mediated immunosuppression.	CRP and soluble CTLA4 are determinants of anti-PD1 resistance in gastrointestinal cancer (96)
	Oncolytic virus (VV–αCTLA-4)	GM-CSF–armed vaccinia virus delivering CTLA-4 antibody intratumorally; depletes Tregs and induces immunogenic tumor cell lysis.	Preclinical: Virus-mediated intratumoral CTLA-4 blockade led to Treg reduction and CD8^+^ T-cell activation, rejecting “cold” tumors.	Vectorized Treg-depleting αCTLA-4 elicits antigen cross-presentation and CD8(+) T cell immunity to reject “cold” tumors (97).
	EGFR/HER2-targeted CAR-T (concept)	CAR-T cells recognizing tumor antigens (independent of MHC); directly lyse tumor cells that evade TCR recognition.	Preclinical/Clinical: EGFR-specific CAR-T showed objective responses in refractory NSCLC; bypasses need for antigen presentation (no direct ref.).	Phase I clinical trial of EGFR-specific CAR-T cells generated by the piggyBac transposon system in advanced relapsed/refractory non-small cell lung cancer patients (98)
T cell-targeted (exhaustion)	CISH–knockout TILs	CRISPR-edited TILs with CISH gene deletion (removing an intracellular brake); enhances TIL proliferation and function.	Clinical (Phase I): In PD-1–refractory GI cancers, CISH-KO TIL therapy was feasible and safe, with one durable complete response observed.	Conversion of unresponsiveness to immune checkpoint inhibition by fecal microbiota transplantation in patients with metastatic melanoma: study protocol for a randomized phase Ib/IIa trial (99)
	High-affinity anti-RGMb mAb	Monoclonal antibody against RGMb (a PD-L2 receptor on T cells); blocks RGMb–PD-L2 interaction to relieve an alternative inhibition pathway.	Preclinical: Anti-RGMb antibody 2C11 restored T cell activity in PD-1–resistant tumor models, highlighting gut microbiome-linked resistance blockade.	Targeting RGMb interactions: Discovery and preclinical characterization of potent anti-RGMb antibodies blocking multiple ligand bindings. MAbs (100)
	LAG-3 or TIGIT antibodies	Checkpoint inhibitors targeting LAG-3, TIGIT, etc.; prevent these inhibitory receptors from sustaining T cell exhaustion.	Clinical: Anti-LAG-3 (relatlimab) + nivolumab improved PFS in melanoma (Phase II/III), leading to approval (Opdualag); Clinical: Anti-TIGIT (tiragolumab) in NSCLC Phase III did not improve survival, indicating target-specific differences (refs. placeholder).	Relatlimab and Nivolumab versus Nivolumab in Untreated Advanced Melanoma (101)
				Tiragolumab plus atezolizumab versus placebo plus atezolizumab as a first-line treatment for PD-L1-selected non-small-cell lung cancer (CITYSCAPE): primary and follow-up analyses of a randomized, double-blind, phase 2 study (102)
Metabolic/other immunomodulators	Taccaoside A (steroidal saponin)	Enhances T-cell metabolic fitness via mTORC1-BLIMP-1; boosts granzyme B secretion and cytotoxicity in T cells.	Preclinical: In ICI-resistant melanoma models, Taccaoside A restored T cell function, leading to tumor regression and improved survival.	Discovery of potent immune-modulating molecule taccaoside A against cancers from structures-active relationships of natural steroidal saponins (103)
	Desaminotyrosine (DAT)	Gut microbial metabolite that boosts type I IFN signaling; primes dendritic cells and NK/T cells, countering dysbiosis effects.	Preclinical: Oral DAT delayed tumor growth and synergized with CTLA-4 blockade in mice; rescued anti-CTLA-4 efficacy after antibiotic-induced microbiome loss.	The microbial metabolite desaminotyrosine enhances T-cell priming and cancer immunotherapy with immune checkpoint inhibitors (120)
	Temozolomide (TMZ)	Alkylating chemotherapy that induces immunogenic cell death; can deplete regulatory T cells at low dose.	Clinical: In PD-1–refractory metastatic melanoma, low-dose TMZ monotherapy yielded anecdotal tumor regressions, attributed to increased tumor antigen release and Treg reduction.	Temozolomide overcoming resistance to immune checkpoint inhibitors in relapsed/refractory metastatic melanoma? Insights from a single center series (104)
Myeloid-targeted	HCK inhibitor (e.g., iHCK)	Inhibits hematopoietic cell kinase in TAMs/MDSCs; reprograms myeloid cells from suppressive to pro-inflammatory phenotype.	Preclinical: In pancreatic cancer models, HCK inhibition reduced fibrosis, increased T-cell infiltration, and overcame anti-PD-1/CTLA-4 resistance.	Inhibition of HCK in myeloid cells restricts pancreatic tumor growth and metastasis (105)
	Anti-Ly6C antibody	Depletes Ly6C^high^ monocytes/blocks their differentiation into suppressive macrophages; prevents accumulation of MDSCs in tumors.	Preclinical: In lung tumor models, anti-Ly6C added to dual PD-1 + CTLA-4 therapy reactivated dendritic cells and cured tumors that had developed resistance.	Targeting immunosuppressive Ly6C+ classical monocytes reverses anti-PD-1/CTLA-4 immunotherapy resistance (106)

**Table 2:** Summary of rational combination therapies to overcome PD-1/CTLA-4 resistance.

Combination strategy	Mechanistic rationale	Key evidence in resistant settings	References
ICI + chemotherapy	Chemo (e.g., oxaliplatin, cyclophosphamide) induces immunogenic cell death (ICD): releases danger signals (HMGB1, ATP) and tumor antigens, increasing dendritic cell activation and TIL infiltration. Some chemotherapies (temozolomide, low-dose cyclophosphamide) also selectively deplete Tregs. These effects recondition the TME, making tumors more responsive to checkpoint blockade.	Preclinical: Oxaliplatin + anti-PD-1 in TNBC models increased calreticulin exposure and CD8^+^ T-cell infiltration, leading to superior tumor control. Clinical: In anti-PD-1–refractory nasopharyngeal carcinoma, PD-1/CTLA-4 bispecific antibody (cadonilimab) plus chemotherapy achieved 68% response rate, vs historical ~20% on chemo alone. In metastatic sarcoma, adding doxorubicin to dual PD-1+CTLA-4 yielded responses in resistant subtypes (33% ORR).	Therapeutic Efficacy of Oxaliplatin and Pembrolizumab Combination Treatment for Triple-Negative Breast Cancer (107)
			Efficacy and safety of cadonilimab (PD-1/CTLA-4 bispecific) in combination with chemotherapy in anti-PD-1-resistant recurrent or metastatic nasopharyngeal carcinoma: a single-arm, open-label, phase 2 trial (108)
			Low-dose metronomic gemcitabine pretreatments overcome the resistance of breast cancer to immune checkpoint therapy (108)
ICI + targeted therapy	Targeted agents block oncogenic or immunosuppressive pathways in tumor cells, removing tumor-intrinsic resistance factors. Examples: VEGF/VEGFR inhibitors normalize vessels and relieve hypoxia; MEK/BRAF inhibitors in melanoma increase tumor antigen expression and T cell infiltration; MET inhibitors reverse immune escape in MET-driven tumors. These changes potentiate ICIs.	Clinical: Pembrolizumab + lenvatinib (VEGFR multi-kinase inhibitor) in PD-1–refractory melanoma improved ORR and OS vs historical controls. Clinical (case): Nivolumab + cabozantinib (MET/VEGFR inhibitor) plus a vaccine induced a durable response in an immunotherapy-resistant sarcoma with a MET fusion. Preclinical: Sphingosine kinase inhibitor (opaganib) added to anti-PD-1 or anti-CTLA-4 enhanced tumor cell ICD and cured tumors in mouse models.	Immunotherapy after progression to double immunotherapy: pembrolizumab and lenvatinib versus conventional chemotherapy for patients with metastatic melanoma after failure of PD-1/CTLA-4 inhibition (109)
			Case report: Robust response of metastatic clear cell sarcoma treated with cabozantinib and immunotherapy (110)
			Opaganib (ABC294640) Induces Immunogenic Tumor Cell Death and Enhances Checkpoint Antibody Therapy (111)
ICI + novel immunomodulator	An emerging class of combinations pairs ICIs with agents that modulate the immune response in ways complementary to checkpoint blockade. These include cytokine therapies (IL-2, IL-12, IL-18 variants) to expand and activate effector T cells; co-stimulatory pathway agonists (CD40, OX40, CD28 agonists) to enhance T cell priming; metabolic enzyme inhibitors (IDO, arginase, adenosine blockers) to relieve immunosuppression; and oncolytic viruses or TLR/STING agonists to inflame “cold” tumors. Each aims to fix a specific immune deficit not addressed by anti-PD-1/CTLA-4 alone.	Clinical: In ICI-progressive melanoma, intratumoral IL-2 led to disease stabilization in some patients and increased CD8^+^ TILs. Decoy-resistant IL-18 (DR-18) plus CTLA-4 blockade boosted effector T cells and reduced Tregs in resistant tumors. Preclinical: CD40 agonist added to radiotherapy + CTLA-4 overcame resistance in mice, but with increased toxicity. An oncolytic adenovirus with TGF-β trap restored anti-PD-L1 responsiveness in resistant models. Clinical: ALPN-202 (CD28 co-stimulator + checkpoint antagonist) showed enhanced T cell activation but caused severe immune toxicity with PD-1 blockade. These illustrate potent synergy but also highlight the need for careful patient selection and safety monitoring.	Targeting immunosuppressive Ly6C+ classical monocytes reverses anti-PD-1/CTLA-4 immunotherapy resistance (106)
			Addition of interleukin-2 overcomes resistance to neoadjuvant CTLA4 and PD1 blockade in ex vivo patient tumors. Sci Transl Med (112)
			Additive Intralesional Interleukin-2 Improves Progression-Free Survival in a Distinct Subgroup of Melanoma Patients with Prior Progression under Immunotherapy (113)
			Decoy-resistant IL-18 reshapes the tumor microenvironment and enhances rejection by anti-CTLA-4 in renal cell carcinoma (114)
			The engineered CD80 variant fusion therapeutic davoceticept combines checkpoint antagonism with conditional CD28 costimulation for anti-tumor immunity (115)

### 4.1 Targeting tumor-intrinsic resistance mechanisms

Tumor-intrinsic resistance often stems from defects in antigen presentation (e.g., loss of MHC class I, B2M loss, JAK1/2 mutations) and oncogenic signaling that creates an immune-“cold” phenotype (116). One approach to counteract this is epigenetic therapy. Histone deacetylase inhibitors (HDACi) can upregulate antigen-processing machinery; for example, entinostat has been shown to increase MHC class I expression and improve anti-PD-1 efficacy in melanoma models (117, 118). Although epigenetic modulators showed promise preclinically, clinical translation has been challenging. The IDO1 enzyme, which depletes tryptophan to impair T cell function, exemplifies this gap: despite strong rationale to combine IDO inhibition with PD-1 blockade, the IDO inhibitor epacadostat failed to improve outcomes in a phase III trial, underscoring the need for robust validation of preclinical targets. Nonetheless, newer epigenetic strategies (e.g., DNA methylation inhibitors, EZH2 blockers) are under investigation to reverse immune silencing in tumors with epigenetic escape mechanisms.

Many oncogenic pathways drive immune evasion—for instance, constitutive β-catenin or EGFR/MET signaling can upregulate PD-L1 and exclude T cell infiltration. Combining targeted inhibitors of these pathways with ICIs can thus overcome resistance. In metastatic melanoma refractory to PD-1/CTLA-4 blockade, adding the multi-kinase inhibitor lenvatinib (targeting VEGFR, FGFR, KIT, and RET) to pembrolizumab improved response rate (23% vs. 11%) and median overall survival (14.2 vs. 7.8 months) compared to chemotherapy. This suggests that inhibiting tumor-intrinsic drivers of an immunosuppressive milieu (e.g., abnormal angiogenesis via VEGF) can re-sensitize tumors to immunotherapy. Similarly, in an aggressive PD-1–resistant sarcoma with an oncogenic c-MET fusion, combining the MET inhibitor cabozantinib with nivolumab (plus a tumor vaccine) induced a durable partial response (119). These cases illustrate a broader principle: rational targeted agents (e.g., EGFR, MET, BRAFV600E, or MEK inhibitors) can reverse tumor-intrinsic immune escape by making tumor cells more visible or vulnerable to the immune system. It is critical to note which of these combinations have advanced clinically—lenvatinib plus pembrolizumab is now being tested in multiple trials, and cabozantinib plus anti-PD-1 has shown activity in certain refractory cancers, whereas other targeted approaches remain exploratory.

Tumors with low mutational burden or poor T cell infiltration may benefit from therapies that introduce new antigens or increase local inflammation. Therapeutic cancer vaccines (e.g., neoantigen vaccines) aim to prime T cells against tumor-specific peptides, potentially overcoming intrinsic “coldness” (120). While personalized vaccines have shown immunogenicity and prolonged relapse-free survival in early trials (e.g., mRNA neoantigen vaccine plus anti-PD-1 in melanoma), their role in overcoming established resistance is still under study. Another strategy is oncolytic virotherapy, which converts immunologically cold tumors into hot ones by infecting tumor cells and releasing tumor antigens along with danger signals. A notable example is a GM-CSF–armed oncolytic vaccinia virus delivering an anti-CTLA-4 payload intratumorally. This virus selectively replicates in the tumor and releases a CTLA-4 antibody (4-E03), which depletes intratumoral Tregs and enhances antigen presentation, achieving tumor regression in preclinical models (97). Oncolytic viruses and localized therapies (including radiotherapy) can thus facilitate antigen cross-presentation and T cell priming in resistant tumors (77). Some are in early clinical testing, but none are yet approved specifically for ICI-resistant disease (121).

An innovative tumor-intrinsic strategy involves eradicating tumor subpopulations that actively drive immune resistance. For instance, tumor cell expression of CTLA-4—and secretion of a soluble form, sCTLA-4—has been shown to mediate resistance to PD-1 blockade in gastrointestinal cancers (122). These CTLA-4^+^ tumor cells not only evade T cell killing but also promote immunosuppression via sCTLA-4. One proposed solution is an antibody-drug conjugate (ADC) targeting CTLA-4 on tumor cells: by delivering a cytotoxic payload specifically to these cells, such an ADC could eliminate the immunosuppressive clone and restore treatment sensitivity. Although still hypothetical, this approach showcases the concept of mechanism-targeted cytotoxic therapy—using precision drug conjugates to eliminate tumor cells that harbor resistance-driving features (e.g., antigen presentation loss or secretion of suppressive factors). As ADC technology advances, similar designs might target, for example, HLA-deficient tumor cells or other “escape” variants, provided a distinguishing surface marker is present. Importantly, any such approach must be guided by biomarker selection in patients whose tumors exhibit the targetable resistance mechanism.

### 4.2 Reversing T cell exhaustion and dysfunction

A central cause of immunotherapy failure is T cell exhaustion, in which tumor-infiltrating lymphocytes become hypofunctional due to chronic antigen exposure and the upregulation of multiple inhibitory receptors (checkpoints). Strategies to reinvigorate these dysfunctional T cells or replace them with competent effectors are therefore critical.

Beyond PD-1 and CTLA-4, several alternative checkpoints on T cells contribute to exhaustion. Therapeutic antibodies targeting LAG-3, TIGIT, and TIM-3 have entered clinical trials, aiming to release these additional “brakes.” Notably, LAG-3 blockade with relatlimab combined with nivolumab was the first such approach to demonstrate improved outcomes (123), achieving a significant progression-free survival benefit in advanced melanoma and leading to regulatory approval in 2022 (Opdualag), thereby validating dual-checkpoint blockade in principle. By contrast, the anti-TIGIT antibody tiragolumab, despite early promise, recently failed to improve survival in a phase II trial of PD-L1 high NSCLC (102), tempering enthusiasm for TIGIT as a standalone target. TIM-3 inhibitors and others (e.g., VISTA, BTLA) remain in early-phase testing. The overall lesson is that co-inhibitory pathways can be heterogeneous across tumors and patients, and blocking a single alternate checkpoint may only benefit specific subsets. Nonetheless, combinatorial blockade (e.g., PD-1 plus LAG-3) can clearly overcome resistance in some cases, and ongoing trials are exploring PD-1 plus TIGIT or TIM-3 in various cancers. Importantly, many of these new agents are still maturing clinically—for example, relatlimab was approved for melanoma (124), and is being evaluated in other tumors (125, 126)—so identifying predictive biomarkers to determine which patients may benefit from an added checkpoint inhibitor is an active area of research.

Another way to reverse T cell dysfunction is to provide activating signals or growth factors to reinvigorate exhausted T cells. High-dose IL-2 was one of the earliest immunotherapies and can expand T cells, but its toxicity limited widespread use. Modern approaches include *engineered cytokines* and *agonists* of T cell costimulatory pathways. For example, adding IL-2 in the context of checkpoint blockade can rescue failing responses: in patients with melanoma progressing on ICIs, intralesional IL-2 led to disease control in a subset, and correlated with increased CD8^+^ TILs (127). Engineered variants like decoy-resistant IL-18 (DR-18) and IL-2/IL-15 hybrids are designed to avoid natural inhibitors and preferentially stimulate effector T cells (114). DR-18 combined with anti-CTLA-4 enriched effector CD8^+^ T cells and reduced Tregs in a resistant tumor model. IL-12, delivered intratumorally or as mRNA, has similarly shown the ability to reactivate “cold” tumors when combined with PD-1/CTLA-4 blockade, promoting a more durable T cell memory response. On the costimulatory side, agonists of CD28 (in a controlled manner) or TNFR family costimulators (such as OX40 and 4-1BB) can enhance T cell activity (128). A novel agent, ALPN-202 (davoceticept), is a fusion protein containing an enhanced CD80 receptor that provides conditional CD28 costimulation when binding PD-L1 on tumor cells, while simultaneously blocking PD-1 and CTLA-4 signals (129). This multipronged approach greatly enhanced T cell activation in preclinical tests. However, initial trials revealed serious toxicity, including fatal myocarditis when combined with pembrolizumab, highlighting that overactivating T cells can be double-edged and must be approached with caution. Still, these strategies exemplify efforts to rescue exhausted T cells by providing them proliferative or costimulatory support, thereby overcoming functional resistance.

When a patient’s endogenous T cells are too exhausted or scarce, another strategy is to replace or supplement them with activated immune cells. Adoptive T cell therapy has shown success in other settings (e.g., CAR-T cells in leukemia) and is being adapted to address resistance in solid tumor immunotherapy. Tumor-infiltrating lymphocyte (TIL) therapy is one approach: TILs are harvested from the patient’s tumor, expanded *ex vivo*, and reinfused. In ICI-resistant melanoma, TIL therapy has induced objective responses in approximately 36% of patients in trials, even when checkpoint blockade failed. To further enhance TIL efficacy, gene editing can be used. A first-in-human study knocked out the *CISH* gene (which encodes an intracellular checkpoint that restrains T cell activation) in TILs (130). These CISH-deficient TILs showed increased reactivity to tumor antigens. In a phase I trial for refractory gastrointestinal cancers, infusion of CISH-knockout TILs was feasible and safe, and one patient achieved a complete response (130). This proof-of-concept demonstrates that engineering T cells to remove inhibitory pathways (here an intracellular negative regulator of cytokine signaling) can overcome T cell-intrinsic resistance. Other cell therapies under study include CAR-T cells targeting solid tumor antigens, as well as NK cell therapies. CAR-T cells can bypass the need for MHC presentation by directly recognizing surface tumor antigens; this could be advantageous in tumors with MHC loss. Preclinical models also suggest that CAR-T or T cell-redirecting bispecific antibodies can eradicate tumor cells that escape T cell recognition (100, 131). For example, bispecific T cell engagers (small antibodies binding a tumor antigen on one arm and CD3 on a T cell with the other) are being developed for solid tumors: by forcibly tethering any T cell to a cancer cell, they trigger T cell killing independent of TCR specificity. Early prototypes (e.g., targeting EpCAM or GP2 in gastrointestinal cancers) have shown tumor regression in mice (132). Clinically, a T cell-redirecting bispecific antibody against GD2 (a melanoma antigen) demonstrated responses in melanoma patients after PD-1 failure, although toxicities were significant. Overall, adoptive cell therapies and engagers provide a means to supply functional immune effectors when the native T cell repertoire is inadequate, and they represent a promising avenue for patients who do not respond even to combined checkpoint blockade. These approaches are mostly in clinical trials or experimental stages; identifying the optimal context (e.g., which resistance mechanism or tumor type) for their use will be key.

Exhausted T cells are also affected by metabolic suppression (e.g., low glucose, high lactate levels) and intracellular inhibitory signals. Thus, drugs that reprogram T cell metabolism or block internal checkpoints may help. One intriguing natural compound is taccaoside A, a steroidal saponin shown to enhance the metabolic fitness of T cells (133). Taccaoside A activates mTORC1 and BLIMP-1 activity in T cells, resulting in greater granzyme B production and cytotoxicity. In ICB-resistant melanoma models, it restored T cell function and eradicated tumors in mice. Though still preclinical, this illustrates how pharmacologically tuning T cell metabolism can reverse functional exhaustion without directly targeting surface checkpoints. Another example is desaminotyrosine (DAT), a metabolite derived from gut microbes. DAT has been shown to promote type I interferon production, which is essential for dendritic cell activation and T/NK cell priming (134). Oral DAT in mice synergized with anti-CTLA-4 therapy and compensated for gut flora loss, as might occur with antibiotic use (135). Such approaches blur the line between targeting T cells and the microenvironment, since metabolites act systemically—yet they ultimately reinvigorate immune cells to fight the tumor. Although compounds like these are far from clinical use, they highlight the wide range of strategies being explored to reverse T cell dysfunction—from blocking inhibitory receptors to fueling T cell activity through metabolic or cytokine support.

### 4.3 Reprogramming the immunosuppressive tumor microenvironment

The tumor microenvironment (TME) in resistant cancers is often hostile to immune attack, being characterized by immunosuppressive cell populations (Tregs, MDSCs, M2 macrophages), inhibitory cytokines and metabolites (TGF-β, adenosine, kynurenine), and physical barriers such as aberrant vasculature. Therapeutic strategies targeting these stromal and microenvironmental factors are critical to overcoming resistance.

Myeloid Cell and Stromal Cell Targets: Tumors often evade immunity by recruiting tumor-associated macrophages (TAMs) and myeloid-derived suppressor cells (MDSCs) that suppress T cells. One emerging approach is to repolarize or deplete suppressive myeloid cells. In preclinical pancreatic cancer, inhibition of HCK (hematopoietic cell kinase), which drives immunosuppressive activation of TAMs, reprogrammed macrophages toward a pro-inflammatory state, reduced fibrosis, and enabled anti-PD-1/CTLA-4 therapy to induce tumor regression (136). Although HCK inhibitors are not yet in the clinic, several CSF-1R inhibitors (which target macrophage survival) have been tested to reduce TAM levels, showing modest activity and are being evaluated in ongoing trials in combination with ICIs. Another strategy is to block factors that recruit or differentiate these cells. For example, anti-Ly6C antibodies prevent monocytes from differentiating into suppressive TAMs/MDSCs; in a lung cancer model, adding anti-Ly6C to dual PD-1 + CTLA-4 blockade overcame acquired resistance by enabling better dendritic cell maturation and T cell priming (106). Targeting cancer-associated fibroblasts (CAFs) is also of interest, as certain CAF subsets foster immunosuppression. In preclinical studies, an antibody against CD73 on a specific pro-tumoral CAF population (CAF-S1) reduced their induction of PD-1^+^CTLA-4^+^ Tregs, and thereby enhanced immunotherapy efficacy. Drugs targeting the adenosine pathway (CD73, CD39, or the A2A adenosine receptor on immune cells) are under investigated, as adenosine in the TME potently inhibits T cell function. Early-phase trials of A2A receptor blockers combined with ICIs have shown some activity in colorectal and lung cancers, although identifying the optimal dose and subset (e.g., adenosine-high tumors) remains critical. Broadly, reprogramming the TME myeloid and stromal components—whether through TAM/MDSC depletion, CAF normalization, or blockade of immunosuppressive metabolites—remains a promising yet complex strategy, with many agents in development and a few (such as CSF-1R inhibitors) in clinical combination trials (97).

Abnormal tumor vasculature and metabolic conditions such as hypoxia also contribute to resistance by excluding immune cells. Anti-angiogenic therapies can normalize blood vessels and improve T cell infiltration. This is one reason why multi-kinase inhibitors (e.g., lenvatinib, cabozantinib) that target VEGF receptors have synergized with ICIs, as discussed above. Normalizing vessels also alleviates hypoxia, which in turn can reduce expression of HIF-1α–driven immunosuppressive genes, such as those that recruit TAMs. Chemotherapy at low or regular doses can similarly modulate these barriers. Metronomic low-dose gemcitabine, for instance, has been shown to normalize tumor stroma and vasculature, increasing T-cell infiltration and sensitivity to PD-1/CTLA-4 blockade in breast cancer models. Likewise, in patients, chemotherapy can “reset” the TME: in refractory nasopharyngeal carcinoma, adding chemotherapy to a PD-1/CTLA-4 bispecific antibody achieved responses in 68% of patients after prior PD-1 failure (137). The concept of immunogenic cell death (ICD) is central here—certain chemotherapies (e.g., oxaliplatin, platinum agents, cyclophosphamide) and targeted agents (such as sphingosine kinase inhibitor opaganib) cause tumor cells to die in a pro-inflammatory manner, releasing ATP, HMGB1, and calreticulin, which attract and activate dendritic cells. This can turn a previously uninflamed tumor into one teeming with antigen-presenting cells and T cells. Clinical trials combining ICIs with ICD-inducing chemotherapies (e.g., oxaliplatin or doxorubicin) have demonstrated improved response rates in resistant cancers such as triple-negative breast cancer and soft tissue sarcoma. Therefore, rational chemotherapy use remains an important TME-targeted strategy, not for its cytotoxic effect alone, but also for its ability to modulate immune contexture (138).

The TME contains soluble factors that dampen immunity, such as TGF-β, IL-10, VEGF, and metabolites including kynurenine (from IDO) and lactate. Therapies that neutralize these factors can lift local immunosuppression. TGF-β traps (e.g., the oncolytic adenovirus AdAPT-001 delivering a TGF-β “trap” protein) have demonstrated that blocking TGF-β in the tumor can restore responsiveness to PD-1 blockade. In a resistant model, AdAPT-001 plus anti-PD-L1 led to tumor regression where anti-PD-L1 alone had failed (139). Similarly, small-molecule inhibitors of the adenosine pathway (CD73 inhibitors or adenosine receptor antagonists) aimed to neutralize adenosine’s immunosuppressive signaling; some are in phase I trials (140). While IDO1 inhibition (epacadostat) was unsuccessful, the tryptophan–kynurenine pathway is still being targeted via upstream (IDO1) or downstream (aryl hydrocarbon receptor) approaches to relieve this brake on T cells. Another promising approach is the microbiome: gut bacteria can influence systemic immunity and response to ICIs. Fecal microbiota transplantation (FMT) from ICI-responsive patients has, in small trials, rescued some refractory melanoma cases—presumably by introducing commensals that produce beneficial metabolites or promote Th1 immunity. A randomized trial of FMT in PD-1-resistant melanoma (NCT05251389) is underway, reflecting the idea that modulating the microbiome (through FMT, probiotics, or microbial metabolites like DAT) could overcome certain resistance states. Indeed, the PD-L2–RGMb pathway discussed earlier exemplifies a microbiome-linked resistance mechanism (RGMb on T cells is regulated by gut flora) that might be targeted through both microbial and antibody-based interventions (141).

Given the complexity of the TME, combination strategies are often required to adequately remodel it. Trials have explored triplet therapies, such as chemotherapy to induce ICD, an ICI to block checkpoints, and an inhibitor or agonist to target a specific TME factor. One example is a regimen tested in intrahepatic cholangiocarcinoma models: gemcitabine/cisplatin chemotherapy was used to normalize vessels and debulk the tumor, CTLA-4 blockade was given to prime T cells, and PD-1 blockade was administered for maintenance. This sequential combination significantly improved survival in an otherwise resistant cancer by activating CXCR3^+^ IFN-γ–producing CD8 T cells and altering the myeloid milieu (142). Ongoing clinical trials are evaluating similar multimodal approaches in challenging indications such as pancreatic cancer and microsatellite-stable colorectal cancer, which are classically unresponsive to single-agent ICIs. The challenge is to maintain tolerability while targeting multiple resistance mechanisms—a subject we discuss further in the *Conclusions*. In sum, targeting the immunosuppressive TME involves a spectrum of tactics: depleting or re-educating suppressive cells, normalizing blood flow and oxygenation, block inhibitory soluble factors, and induce pro-immunity cell death. Each has shown signs of success, and the most effective regimens will likely integrate several of these in a patient-specific manner.

## 5 Predictive biomarkers and machine learning

Identifying patients who will not respond to PD-1/CTLA-4 inhibitors—or who will relapse after an initial response—is crucial for guiding the above therapeutic strategies. A growing array of predictive biomarkers and machine learning (ML)-based signatures is being developed to foresee immunotherapy resistance. These tools can help identify patients for intensified or alternative therapies *before* clinical resistance manifests, thereby personalizing treatment. In this section, we summarize key advances in predictive modeling and biomarkers (**Table 3** in the data compendium) and discuss how they integrate with therapeutic decision-making.

**Table 3:** Predictive signatures and biomarkers associated with primary or acquired resistance to immune checkpoint inhibitors (ICIs).

Category		Predictive method/feature	Key findings	Reference
Machine learning-driven signatures	Immune-Related Exosome Signature (IES)	RSF + Elastic Net model (C-index = 0.75)	High IES score correlates with immune exclusion, high TIDE score, and low PD-1/CTLA-4 immunophenoscore.	Machine learning developed immune-related exosome signature for prognosis and immunotherapy benefit in bladder cancer (143)
	Stemness-Related Score (SRscores)	11-gene random forest model	High SRscores predict poor survival and resistance to anti-PD-1/anti-CTLA-4 therapy in HCC	Deep dissection of stemness-related hierarchies in hepatocellular carcinoma (144)
	NK Cell-Related Signature	LASSO + CoxBoost model (11 genes)	Low-risk group identified by this signature shows higher PD-1/CTLA-4 immunophenoscore and improved ICI response in HCC	Combining bulk and single-cell RNA-sequencing data to develop an NK cell-related prognostic signature for hepatocellular carcinoma based on an integrated machine learning framework (145)
	M2-like Macrophage Signature (MRPS)	Stepwise Cox + SuperPC algorithm	Low MRPS is associated with activated CD8+ T cells and NK cells, and better immunotherapy response in HCC	Identification of M2-like macrophage-related signature for predicting the prognosis, ecosystem and immunotherapy response in hepatocellular carcinoma (146)
	Ubiquitin Score	Quantitative ubiquitin-modification score	High ubiquitin score correlates with an inflamed TME, elevated PD-1/PD-L1/CTLA-4 expression, and sensitivity to anti-PD-1 therapy	Ubiquitin Modification Patterns of Clear Cell Renal Cell Carcinoma and the Ubiquitin Score to Aid Immunotherapy and Targeted Therapy (147)
	m6Ascore	PCA-derived N6-methyladenosine (m6A) score	Low m6Ascore corresponds to an immune-inflamed tumor phenotype (~90% ICI response) vs. high m6Ascore indicates immune-exclusion (~10% ICI response) in melanoma	N6-methyladenosine RNA Methylation Correlates with Immune Microenvironment and Immunotherapy Response of Melanoma (148)
	Intratumor Heterogeneity (ITH) Signature	LASSO-derived heterogeneity score	Low ITH signature score predicts better survival, lower TIDE score, and higher PD-1/CTLA-4 immunophenoscore in melanoma	Machine learning developed an intratumor heterogeneity signature for predicting prognosis and immunotherapy benefits in skin cutaneous melanoma (149)
	NFATC2 Transcriptional Signature	NFATC2 target gene expression signature	High NFATC2 signature score is associated with resistance to ICI therapy: reduced CD8+ T-cell infiltration and T-cell exhaustion in melanoma	NFATC2 target gene signature correlates with immune checkpoint blockade resistance in melanoma (150)
Clinical biomarkers	Peritoneal Metastases with Ascites	–	Presence of peritoneal metastases with ascites predicts primary resistance to anti-PD-1 ± anti-CTLA-4 therapy in dMMR/MSI-H metastatic colorectal and gastric cancers; linked to an immunosuppressive TME	Ascites and resistance to immune checkpoint inhibition in dMMR/MSI-H metastatic colorectal and gastric cancers (151)
	Pan-Immune-Inflammation Value (PIV)	(Neutrophils × platelets × monocytes) / lymphocytes	High baseline PIV (>492) and early increase (≥30%) predict poor OS and PFS in MSI-H metastatic colorectal cancer patients on ICIs	The Pan-Immune-Inflammation Value in microsatellite instability-high metastatic colorectal cancer patients treated with immune checkpoint inhibitors (152)
	Soluble TIM-3 (sTIM-3)	Soluble T-cell immunoglobulin mucin-3	Elevated baseline sTIM-3 in plasma (metastatic clear cell RCC) predicts lack of response to anti-PD-1 monotherapy (but not combination), guiding therapy selection	Soluble TIM-3, likely produced by myeloid cells, predicts resistance to immune checkpoint inhibitors in metastatic clear cell renal cell carcinoma (153)
	Soluble CD27 (sCD27)	Soluble CD27 (T-cell co-stimulatory receptor)	High baseline sCD27 predicts anti-PD-1 monotherapy failure in melanoma, suggesting benefit from upfront combination (anti-PD-1 + anti-CTLA-4) therapy	Soluble CD27 differentially predicts resistance to anti-PD1 alone but not with anti-CTLA-4 in melanoma (154)
	Serum Metabolomic Profile	Lactate, tryptophan, histidine, proline levels	Pretreatment serum metabolite levels (e.g., low lactate, high histidine/proline/tryptophan) are associated with longer overall survival in metastatic melanoma patients on combined anti-CTLA-4 + anti-PD-1 therapy	Metabolomic signatures in liquid biopsy are associated with overall survival in metastatic melanoma patients treated with immune checkpoint inhibitor therapy (155)
	Soluble LAG-3 (sLAG3)	Soluble LAG-3 (checkpoint receptor)	High baseline sLAG-3 (>377 pg/mL) independently predicts shorter PFS and OS in advanced HNSCC, consistent with ICI resistance	The Role of Soluble LAG3 and Soluble Immune Checkpoints Profile in Advanced Head and Neck Cancer: A Pilot Study (156)
	Prior Chemotherapy History	Prior exposure to chemotherapy	History of prior chemotherapy is associated with reduced ICI response rates in NSCLC; however, patients with high TMB (≥15 mut/Mb) show preserved ICI efficacy, mitigating this resistance factor	High tumor mutation burden mitigates the negative impact of chemotherapy history on immune checkpoint blockade therapy (157)

### 5.1 Multi-omics signatures and machine learning models

High-dimensional molecular data from tumors (e.g., gene expression, epigenetics) can reveal patterns associated with immunotherapy outcomes. Machine learning-driven signatures have emerged as powerful predictors by integrating these complex features. For example, Luo et al. built an immune-related exosome signature (IES) for bladder cancer using a hybrid ML approach (random survival forest plus elastic net), achieving a C-index of approximately 0.75 for survival prediction (143). Patients with a high IES had immunologically “cold” tumors (T-cell excluded, high dysfunction scores), correlating with resistance to PD-1/CTLA-4 therapy. In hepatocellular carcinoma, a machine-learned stemness-related score (SRscore) based on 11 gene expressions stratified patients by outcome: those with high stemness signatures had significantly poorer survival and showed non-response to ICIs (144). Similarly, an NK cell-oriented gene signature constructed via LASSO plus CoxBoost in HCC could predict which tumors had a permissive environment for ICIs, as low-risk groups (indicative of robust NK/T cell presence) showed better responses (145). Across cancer types, dozens of such ML-derived signatures now exist, capturing facets such as intratumor heterogeneity (ITH), immune cell infiltration profiles, and specific transcriptional programs (e.g., an NFATC2-driven T cell exhaustion signature (150)). These scores are typically validated against known correlates of response: for instance, patients classified as low-risk by an ML model (158) consistently show higher baseline immunogenicity (high PD-L1, CD8^+^ T cells, interferon-γ signature) and lower TIDE scores (Tumor Immune Dysfunction and Exclusion algorithm). Conversely, high-risk patients often have biomarkers of resistance, such as T cell exclusion, elevated Tregs or M2 macrophages, and activation of wound-healing or stemness pathways. The upshot is that ML can distill these complex variables into a single predictive index (such as an “ICB resistance score”). In practice, these signatures could guide therapy by flagging patients unlikely to respond to standard ICIs: such patients might be triaged to upfront combination therapy (to preempt resistance) or to alternative treatments. However, most ML signatures are still retrospective; prospective trials are needed to test whether using them to assign therapy improves outcomes. The integration of ML into clinical practice also faces challenges of reproducibility and data standardization, but it holds great promise for sharpening predictive capabilities beyond simpler markers such as PD-L1 expression or tumor mutational burden.

### 5.2 Clinical and liquid biopsy biomarkers

Alongside computational signatures, more practical clinical biomarkers are being recognized. Some are clinical features or routine laboratory values that correlate with resistance. A striking example is the site of metastasis: the presence of peritoneal metastases with ascites strongly predicts primary resistance to ICIs in certain cancers. In metastatic MSI-high colorectal and gastric cancer, patients with malignant ascites had significantly worse responses and survival on PD-1 ± CTLA-4 antibodies than those without ascites (159). The ascites likely indicate a highly immunosuppressive TME (rich in inhibitory cytokines and myeloid cells), suggesting these patients may require more aggressive combination approaches from the start. Another easily measured metric is systemic inflammation. The Pan-Immune-Inflammation Value (PIV), a composite index of neutrophil, platelet, monocyte, and lymphocyte counts, has been shown to predict ICI outcomes. In MSI-high colorectal cancer, a high baseline PIV (>492) was associated with significantly shorter progression-free survival and overall survival on PD-1 blockade (152). Moreover, an early rise in PIV after starting therapy predicted resistance. These findings echo earlier observations that a high neutrophil-to-lymphocyte ratio and elevated CRP (C-reactive protein) correlate with poor ICI responses in various cancers. Interestingly, CRP has mechanistic ties to resistance: inflammation-driven CRP can induce tumor CTLA-4 expression and sCTLA-4 production, as described earlier, thereby blunting T cell activation (96). Thus, systemic inflammatory markers not only prognosticate outcomes but also reflect biological processes antagonistic to immunotherapy, such as neutrophil- or CRP-mediated immunosuppression.

Soluble immune checkpoint proteins measured in blood are another emerging class of biomarkers. These represent shed or secreted forms of checkpoint receptors or ligands. For instance, soluble TIM-3 (sTIM-3) in the plasma of patients with metastatic renal cell carcinoma was found to predict anti-PD-1 failure (160). Patients with high sTIM-3 had poor responses to nivolumab, whereas interestingly, those same patients could still respond when nivolumab was combined with ipilimumab (anti-CTLA-4). This suggests that sTIM-3 might identify individuals who require dual checkpoint blockade rather than PD-1 monotherapy. Similarly, soluble CD27, a marker of T cell activation, was shown to predict melanoma outcomes: high baseline sCD27 correlated with lack of benefit from PD-1 monotherapy, whereas those patients did well when CTLA-4 was added (154). It appears that high levels of certain soluble checkpoints (e.g., TIM-3, CD27, LAG-3) signal an immune environment that is too suppressed for single-agent PD-1 to succeed, thereby pointing to combination treatment. In head and neck cancer, elevated soluble LAG-3 was associated with shorter survival on ICIs (156). These soluble factors can be considered liquid biopsy correlates of T cell exhaustion or tumor immune evasion, they likely reflect high intratumoral expression of these checkpoints and an ongoing T cell–tumor struggle. If validated, these markers could be measured before treatment to stratify patients. For example, a melanoma patient with very high sLAG-3 or sCD27 might be directed toward nivolumab plus ipilimumab up front, whereas a patient with low levels might suffice with PD-1 monotherapy.

Finally, metabolomic and genomic biomarkers are being explored. Pretreatment serum metabolite profiles (e.g., high lactate and low tryptophan) have been associated with early progression on ICIs in melanoma (155). Metabolites can reflect both tumor and host metabolic states (such as cachexia or microbiome activity) that influence immunity. On the genomic side, ultra-high tumor mutational burden (TMB) is a well-established positive predictor for ICI response; interestingly, recent data suggest TMB can modulate other predictors. One study in lung cancer found that prior chemotherapy exposure usually diminished ICI efficacy, except in patients with very high TMB, where responses remained robust despite chemotherapy history (157). This finding implies that genomic features such as TMB can sometimes offset negative clinical factors, potentially by indicating a strongly immunogenic tumor capable of overcoming therapy-induced immune damage. Additionally, specific genomic alterations (e.g., STK11/LKB1 mutations in lung adenocarcinoma) are known to confer primary resistance to PD-1 blockade; these findings could guide patients to alternative approaches (such as combining ICIs with metabolic or epigenetic agents to counteract the mutation’s effects).

In summary, the field of predictive biomarkers is rapidly expanding. We now have integrative ML models that condense tumor molecular data into a “resistance score,” as well as simpler biomarkers—from site of metastasis and blood counts to soluble proteins and metabolite levels—that can be measured in the clinic. The ultimate vision is to combine these predictors into a robust decision algorithm: for each patient, assess tumor PD-L1, TMB, an ML-derived gene signature, and key clinical biomarkers (e.g., ascites, PIV, sCD27). If the composite indicates a high risk of resistance, clinicians might start with a combination of therapies targeting the likely mechanisms (e.g., adding an anti-TIGIT or an IDO inhibitor, or using chemo-ICI upfront). If the risk is low, patients could proceed with standard ICI monotherapy, thereby sparing them unnecessary toxicity. Machine learning–guided personalization of immunotherapy is still in its early stages, but it represents a crucial companion to the development of new treatments—ensuring that the right patients receive the right combination at the right time.

## 6 Discussion

Resistance to PD-1 and CTLA-4 blockade remains a formidable obstacle, but significant progress has been made in deciphering its multifaceted biology. This knowledge is actively fueling a pipeline of innovative therapeutic strategies—ranging from next-generation mono-immunotherapies and bispecific antibodies to rational combinations targeting the tumor microenvironment and tumor-intrinsic escape pathways. A recurring theme is that effective interventions often need to address multiple resistance mechanisms simultaneously. Clinical trials combining ICIs with chemotherapy or targeted agents (e.g., anti-angiogenics such as lenvatinib) have already shown improved outcomes in ICI-refractory cancers, validating the principle of mechanism-guided synergy. Looking ahead, the integration of advanced biomarkers and machine learning is poised to optimize these strategies: robust predictive signatures (incorporating genomic, immunologic, and clinical features) will help identify patients who require upfront combination therapy or novel agents, versus those likely to respond to standard therapy alone (115). Such personalization is key to maximizing therapeutic benefit while minimizing unnecessary toxicity.

Challenges certainly remain. Many of the discussed approaches are in early development, and translating preclinical successes into safe, effective treatments remains challenging. Trial design will need to carefully balance efficacy with toxicity, especially as multiple immune-active agents are combined. Immune-related adverse events (irAEs) can be severe—for example, combining checkpoint blockade with T cell co-stimulatory agonists resulted in cases of fatal myocarditis and colitis (115). This underscores that more intensive immunotherapy must be paired with vigilant toxicity monitoring and prompt management protocols. Research into biomarkers for irAE risk is also crucial, enabling the identification of high-risk patients who may benefit from prophylactic measures or alternate regimens. Moreover, tumor heterogeneity means that no single strategy will overcome resistance in all cases; thus, a continued emphasis on combination approaches and adaptive treatment strategies (e.g., adding therapies at signs of resistance) will be important (115, 130).

In conclusion, the fight against immunotherapy resistance is rapidly evolving. Our growing understanding of tumor–immune interactions—from antigen presentation and T cell biology to the influence of the microbiome and host factors—is being translated into innovative therapeutic interventions. Through mechanism-guided combinations and personalized treatment selection, it is becoming increasingly feasible to convert previously “immune-cold” tumors into responsive ones. The ultimate goal is to extend the remarkable, durable remissions seen in immunotherapy responders to a much larger fraction of patients. By tackling resistance on multiple fronts and learning from each setback (such as negative trials of IDO inhibition), the field is moving closer to that goal. What was once an intractable endpoint—resistance—is now viewed as a challenge to be understood and overcome, heralding a future in which long-term cancer control via immunotherapy is achievable for many more patients.
